# Antimicrobial peptide temporin derivatives inhibit biofilm formation and virulence factor expression of *Streptococcus mutans*

**DOI:** 10.3389/fmicb.2023.1267389

**Published:** 2023-09-26

**Authors:** Shangjun Jiang, Yanmei Zha, Ting Zhao, Xiao Jin, Ruiying Zhu, Shuangshuang Wei, Rong Wang, Yanting Song, Lushuang Li, Junchen Lyu, Wenting Hu, Daqi Zhang, Manchuriga Wang, Yingxia Zhang

**Affiliations:** ^1^Key Laboratory of Tropical Biological Resources of Ministry of Education, School of Pharmaceutical Sciences, Collaborative Innovation Center of One Health, Hainan University, Haikou, China; ^2^College of Life Sciences, Hainan University, Haikou, China; ^3^School of Science, Hainan University, Haikou, China; ^4^Department of Neurology, The First Affiliated Hospital of Hainan Medical University, Haikou, China; ^5^School of Animal Science and Technology, Hainan University, Haikou, China

**Keywords:** antimicrobial peptides, temporin, *Streptococcus mutans*, antibiofilm, anti-caries

## Abstract

**Introduction:**

Temporin-GHa obtained from the frog *Hylarana guentheri* showed bactericidal efficacy against *Streptococcus mutans*. To enhance its antibacterial activity, the derived peptides GHaR and GHa11R were designed, and their antibacterial performance, antibiofilm efficacy and potential in the inhibition of dental caries were evaluated.

**Methods:**

Bacterial survival assay, fluorescent staining assay and transmission electron microscopy observation were applied to explore how the peptides inhibited and killed *S. mutans*. The antibiofilm efficacy was assayed by examining exopolysaccharide (EPS) and lactic acid production, bacterial adhesion and cell surface hydrophobicity. The gene expression level of virulence factors of *S. mutans* was detected by qRT-PCR. Finally, the impact of the peptides on the caries induced ability of *S. mutans* was measured using a rat caries model.

**Results:**

It has been shown that the peptides inhibited biofilm rapid accumulation by weakening the initial adhesion of *S. mutans* and reducing the production of EPS. Meanwhile, they also decreased bacterial acidogenicity and aciduricity, and ultimately prevented caries development in vivo.

**Conclusion:**

GHaR and GHa11R might be promising candidates for controlling *S. mutans* infections.

## Introduction

Dental caries adversely affects more than 60% of school-aged children and most adults worldwide and has been identified as the third major disease endangering human health by the World Health Organization (WHO) after cardiovascular diseases and malignant tumors (Peres et al., 2019). When oral microorganisms attach to tooth surfaces, an acquired biofilm is formed, mainly composed of salivary glycoproteins. Subsequently, the initially established weak interaction between bacterial adhesins and cell membrane glycoprotein receptors became stronger, followed by more bacteria aggregating to the attached bacteria, forming multispecies communities, known as dental plaque biofilms. Biofilms are constructed by highly organized oral microorganisms and are rich in proteins, extracellular polysaccharides (EPSs), carbohydrates, and nucleic acids, providing a perfect extracellular polymer matrix for the subsequent adhesion of oral cariogenic bacteria, in some cases leading to the progression of oral biofilms into cariogenic biofilms (Mosaddad et al., 2019). Among the oral bacteria, *Streptococcus*, *Actinomyces*, and *Lactobacilli* contribute more to secreting glucan to cell surfaces, thereby strengthening the adhesion of cariogenic microorganisms to teeth. At the same time, they ferment carbohydrates into lactic acid, which continuously demineralizes teeth and leads to the tooth decay (Ma et al., 2015; Lemos et al., 2019).

*Streptococcus mutans* is considered to be the dominant etiologic bacteria in dental caries (Lemos et al., 2019) and a potential risk factor in cardiovascular diseases, as it has been detected on cardiac valves and atherosclerotic plaques in patients (Nakano et al., 2006). *Streptococcus mutans* secretes a large amount of lactic acid synthesized by lactate dehydrogenase (LDH) in the presence of dietary sugars in oral cavity, causing serious damage to dental hard tissues (Ma et al., 2022). Most bacteria have difficulty growing in a low pH environment created by lactic acid, but *S. mutans* actively pumps protons out of the cells by consuming ATP to maintain intracellular pH and ensure growth, which leads to remarkable aciduricity (Harper and Loesche, 1983). In addition, *S. mutans* can synthesize extracellular polysaccharides (EPS) using dietary sucrose by its glucosyltransferases (GTFs) and fructosyltransferases (FTFs) (Zhang et al., 2021) and secrete them onto cell surfaces, enhancing the adhesion ability of *S. mutans*; meanwhile, they also provide a matrix for colonization of other cariogenic bacteria, leading the dental plaque biofilm formation (Klein et al., 2015). The microorganisms embedded in biofilms exhibit low metabolic activities and strong tolerance to antimicrobial reagents (Yue et al., 2018). Therefore, targeting *S. mutans* has become an effective way to prevent the onset of dental caries or treat early caries.

Antimicrobial peptides (AMPs) with broad-spectrum activities against pathogenic microorganisms have widely found in natural organisms, which are important components in the body’s innate immune system and produced by almost all tissues and cells that are regularly exposed to microorganisms (Wiesner and Vilcinskas, 2010). The oral cavity is an open environment, in which the microbiome is acquired in childhood and will fluctuate in the proportion of species by such as diet and age. Once the homeostasis is broken, the pathogenic microorganisms might be predominant and cause oral diseases (Diamond et al., 2008). Several antimicrobial peptides, including statin, defensin, and LL-37, were founded in the oral epithelium and saliva and were considered to be the core defense system in the oral cavity (Guncu et al., 2015). Additionally, known as host defense peptides, these naturally occurring AMPs in the oral cavity are involved in regulating the innate immune response of host cells and exhibit resistance to oral pathogenic microorganisms (Griffith et al., 2022).

Temporin peptides, first found in amphibians, are one of the shortest naturally occurring peptide families, consisting of 10–14 amino acid residues. Because of their low manufacturing cost and diverse activities, these peptides are ideal candidates for therapeutic drug development (Wang et al., 2016). Through previous studies, we cloned natural antimicrobial peptide temporin-GHa (GHa) (FLQHIIGALGHLF-NH_2_) (Dong et al., 2017). In this article, we designed temporin-GHa11R (GHa11R) (FLQHIIGALGRLF-NH_2_) and temporin-GHaR (GHaR) (FLQRIIGALGRLF-NH_2_) to improve the antimicrobial and antibiofilm activities under physiological conditions by enhancing the net positive charges of GHa. The bacteriostatic effects and mechanism of action of the derived peptides were measured *in vitro*. The anti-caries properties of GHaR and GHa11R were studied in the caries rat model induced by *S. mutans*.

## Materials and methods

### Peptides, bacterial strains, and growth conditions

The peptides were synthesized by Ji’er Biochemical (Shanghai, China) with a purity greater than 95%. Before use, the peptides were dissolved in sterile deionized water at the concentration of 2 mM as stock solution, and diluted with phosphate buffered saline (PBS) to work concentration. *Streptococcus mutans* UA159 was purchased from Guangdong Microbial Culture Collection Center (Guangzhou, China) and cultured anaerobically in brain heart infusion broth (BHI; Thermo Fisher, CN) at 37°C under mixed gas conditions of 5% CO_2_, 10% H_2_, and 85% N_2_ to logarithmic growth phase before experiments. The colony forming units (CFUs) and the optical density at 600 nm (OD_600_) of the bacteria were plotted. OD_600_ = 0.1 is equal to the density of *S. mutans* at 2 × 10^6^ CFU/mL in BHI.

*Streptococcus sanguis* ATCC 10556 and *Porphyromonas gingivalis* ATCC 33277 were cultured anaerobically in BHI. *Bifidobacterium adolescentis* ATCC 15703 and *B. breve* ATCC 15700 were cultured anaerobically in MRS broth (MRS; Thermo Fisher, CN) containing 0.05% L-cysteine hydrochloride. *Lactobacillus acidophilus* ATCC 4356 was cultured anaerobically in MRS broth.

### Peptide structure determination by circular dichroism (CD) spectroscopy

The peptides (1 mM) were dissolved in 10 mM PBS, 30 mM sodium dodecyl sulfate (SDS) and 50% (v/v) trifluoroethanol (TFE), respectively. The peptide spectrum was scanned by a CD spectrometer (Brighttime Chirascan, Applied Photophysics Limited, UK) at wavelengths of 190 nm to 260 nm with a speed of 10 nm/min.

### Antibacterial activity assay

Equal volumes (50 μL) of bacterial suspension (2 × 10^6^ CFU/mL) and the peptide solution were mixed in wells on polystyrene plates (Corning, USA). After the plates were incubated anaerobically for 24 h, the OD_600_ was measured. In negative control group, the peptides were replaced with PBS only, and chlorhexidine (CHX) was used as a positive control. The minimum inhibitory concentration (MIC) is defined as the minimum concentration that inhibits the visible growth of bacteria within 24 h. The minimum bactericidal concentration (MBC) is the minimum concentration that kills 99% of bacterial cells determined by colony counting (Cao et al., 2020).

### Time killing assay

The bacterial suspension (2 × 10^6^ CFU/mL, 100 μL) in BHI was mixed with the same volume of the peptide solution (3.1–12.5 μM). The peptides were replaced with PBS only in the negative control. Aliquots of the mixtures were pipetted at 0, 15, 30, 45, 60, 90, 120, and 180 min, and plated on BHI agar after proper dilution. The plates were incubated anaerobically for 48 h, followed by colony counting.

### Observation of bacteria by transmission electron microscopy (TEM)

After exposing to the peptides (4 × MIC) for 1 h, the bacteria (1 × 10^9^ CFU/mL) were collected, fixed with 2.5% glutaraldehyde and dehydrated in serially diluted ethanol solutions at room temperature. The peptides were replaced with PBS only in the negative control. After embedding in resin for 48 h at 60°C, the bacteria were sliced into 60–80 nm sections and loaded on 150 mesh copper mesh. After staining with 2% uranium acetate, the sections were washed with 70% ethanol solution and ultra-pure water, respectively, then re-stained with 2.6% lead citrate solution. After washing and drying overnight, the sections were observed by TEM (HT7800, Hitachi, Japan) (Zhang et al., 2022).

### Cell membrane integrity assay

The cell membrane integrity was studied by fluorescent staining (Zhang et al., 2020). *Streptococcus mutans* (1 × 10^9^CFU/mL) pre-treated with the peptides (1 × MIC) for 1 h was centrifuged and collected to remove the peptides. The bacteria were fixed with 4% paraformaldehyde for 1 h, followed by staining with PI solution (10 μg/mL) for 15 min in the dark. Then the bacteria were collected by centrifugation and stained with DAPI solution (10 μg/mL) for 15 min. The bacteria were washed, collected, and observed under an inverted fluorescence microscope with *S. mutans* treated with PBS as a negative control.

To confirm the impact of the peptides on *S. mutans* cell membranes, *S. mutans* treated with the peptides and stained with PI as described above were detected by flow cytometry (BF32366, Beckman Coulter, Brea, CA, USA).

### Glycolytic pH drops and acid tolerance assay

Influence of the peptides on acid-producing capacity of *S. mutans* has been assessed (Wang et al., 2018). The bacterial suspension was adjusted to 1 × 10^8^ CFU/mL in BHI broth (the initial pH was 7.2) containing the peptides (1/2 × MIC, at which the bacterial growth was not affected), and 1% (w/v) glucose was added to trigger glycolysis. In negative control, the peptides were replaced with PBS only. After incubating for 180 min, an aliquot of the suspension was sampled every 30 min for detecting the pH value using a glass electrode pH meter.

Acid tolerance of *S. mutans* was assessed (Svensater et al., 1997). Bacteria (1 × 10^6^ CFU/mL) grown in tryptone-yeast extract medium (TYEM) broth (pH 7.5, containing 20 mM glucose) were transferred to BHI culture medium (pH 5.5) with or without the peptides (1/2 × MIC). The same volume of suspension was diluted and applied to BHI agar for determining the number of viable bacteria. After the remaining suspension was incubated for 2 h, the pH was immediately reduced to the killing pH (3.0) for *S. mutans* by adding 20% hydrochloric acid and further incubated for 3 h. The suspensions were also plated. After incubating all plates anaerobically for 48 h, the total CFUs were calculated.

### Lactic acid production assay

The bacterial suspensions (1 × 10^6^ CFU/mL, 2 mL) were added to each well containing a coverslip on a 24-well microtiter plate, and incubated anaerobically for 24 h. After discarding planktonic bacteria, the coverslips covered by biofilms were removed to a new 24-well plate, and treated by the peptides (1/2 × MIC, containing 2% sucrose) in buffered peptone water (BPW). In negative control, the peptides were replaced with PBS only. After incubating anaerobically for 180 min, the supernatant was collected, and the production of lactic acid was measured by lactic acid measurement kit (Solarbio, China). OD_570_ was recorded and the concentration of lactic acid was calculated (Chen et al., 2020).

### Cell membrane permeabilization and F_1_F_0_-ATPase assay

The permeabilized cells were prepared and the activity of F_1_-F_0_ ATPase was determined (Xu et al., 2011). In general, *S. mutans* was collected and dispersed in Tris–HCl buffer (pH 7.0) containing 10 mM MgSO_4_ and 10% (v/v) toluene. After swirling for 1 min, the bacterial suspension was incubated for 5 min at 37°C and rapidly frozen in liquid nitrogen. The frozen cells were quickly thawed and re-frozen twice. After collecting and exposing to the peptides (1/2 × MIC) for 120 min, the bacteria were collected by centrifugation and resuscitated in Tris-maleate buffer (pH 7.0, containing 10 mM MgCl_2_). Finally, 5 mM ATP was added to trigger F_1_-F_0_ ATPase activity. In negative control, the peptides were replaced with PBS only. The released phosphate was identified (Bencini et al., 1983).

### The assay of inhibition biofilm formation

The effect of the derived peptides on the biofilm formation of *S. mutans* was evaluated (de Breij et al., 2018). Briefly, an equal volume of bacterial suspension (2 × 10^6^ CFU/mL) in BHI broth containing 1% sucrose (BHIs) was mixed with the peptide solution (0.8 to 25 μM) on a 96-well polystyrene plate (Corning, USA), and incubated anaerobically for 24 h. In negative control, the peptides were replaced with PBS only. After discarding the supernatant, the remaining biofilm was rinsed twice with PBS, fixed by methanol solution and thoroughly dried. CV solution (0.1%) was added to stain the biofilm for 15 min. After the excess dye was thoroughly washed with PBS, 200 μL of ethanol was added and OD_595_ was determined.

### Bacterial adhesion assay

The activity of the derived peptides on the bacterial adhesion ability was evaluated both on 96-well polystyrene plate surfaces and saliva-coated surfaces (Zhang et al., 2020). Bacterial suspension (1 × 10^9^ CFU/mL) was treated with the peptides (4 × MIC) for 15 min and the unbound peptides were removed by centrifugation (3,000 × *g*, 3 min). In negative control, the peptides were replaced with PBS only. After resuspending in BHI broth, an aliquot of the suspension was pipetted, plated on BHI agar, and cultured anaerobically for 48 h. The CFUs were calculated to determine the number of viable bacteria. A 96-well polystyrene plate was precoated with 100 μL of artificial saliva at 37°C for 15 min. After the saliva was removed, the saliva-coated plate was prepared. The remaining suspension (100 μL) was added to a polystyrene plate or a saliva-coated plate and incubated for 1 h under static conditions. After discarding the supernatant, the plates were gently washed twice with PBS to remove unadhered bacteria. After adding 200 μL of PBS to each well, all the plates were sonicated three times at a power of 120 W and a frequency of 40 kHz on ice for 90 s with an interval of 30 s. The sonication fluid was plated and the CFUs were numbered. The bacterial adhesion rate was expressed as the CFUs of adhesive bacteria divided by the CFUs of the viable bacteria.

### Cell surface hydrophobicity assay

The bacterial suspension (1 × 10^9^ CFU/mL) was incubated with the derived peptides (0.8–25 μM) in Phosphate urea magnesium (PUM) buffer for 20 min, and the OD_550_ of the bacterial suspension was measured. Cetane (200 μL) was added to the remaining bacterial suspension, vortexed, and settled for 30 min. In negative control, the peptides were replaced with PUM only. The supernatant was pipetted to determine the OD_550_. The degree of changes in surface hydrophobicity of *S. mutans* was shown as a percentage decrease of the OD_550_ (Kim et al., 2019).

### Water-insoluble EPS measurement

The production of water-insoluble EPS in biofilms was evaluated by phenol-sulfuric acid method (Abdel-Aziz et al., 2020). Briefly, equal volumes of bacterial suspension (1 × 10^6^ CFU/mL) and the peptides (1 × MIC) were mixed in a 24-well polystyrene plate and incubated anaerobically for 24 h to generate biofilms. In negative control, the peptides were replaced with PBS only. After discarding planktonic bacteria, the biofilms were collected and washed twice to clean out water-soluble EPS. After centrifugation, the pellet was re-suspended in 1 M NaOH solution (200 μL) for 2 h to extract water-insoluble EPS. Finally, 5% frozen phenol and sulfuric acid were added to the mixture at 1:1:5 (volume ratio), incubated for 1 h, followed by measuring OD_625_.

### Confocal laser scanning microscope (CLSM) observation

The EPS distribution in biofilms was observed by CLSM (Deng et al., 2021). Equal volumes (1 mL) of the bacterial suspension (2 × 10^6^ CFU/mL) in BHIs broth and the peptides solution (1 × MIC) were mixed in each well containing a coverslip on a 24-well polystyrene plate. In negative control, the peptides were replaced with PBS only. Alexa Fluor 647 glucan (1 μmol/L) was used to label the dextran conjugate for 24 h. After that, *S. mutans* nucleic acids were labeled with SYTO 9 (2.5 μmol/L). CLSM (TCS SP8, LEICA, Germany) was used to observe.

### The expression of virulence genes by quantitative real-time reverse transcription PCR (qRT-PCR)

The expression levels of virulence genes in *S. mutans* were measured by qRT-PCR with 16S rRNA serving as a quantified internal control (Xiong et al., 2020; Aqawi et al., 2021). *Streptococcus mutans* in the logarithmic growth phase was diluted to 1 × 10^6^ CFU/mL in BHIs broth, pretreated with the peptides (1/2 × MIC, diluted with BHIs) for 8 h and total RNA was purified. In negative control, the peptides were replaced with PBS only. The first-strand cDNA was synthesized using RT kit (TES201, Tsingke, China). The tested genes and primers are listed (Table 1). The reaction was performed on a CFX96 real-time system (CFX Connect, Bio-Rad, USA). Gene expression was presented by using the *2*^–Δ Δ^ *^Ct^* method and normalized to the 16S rRNA level.

**TABLE 1:** The primers used for quantitative real-time reverse transcription PCR.

Gene	Forward primer	Reverse primer
*16S* *RNA*	AGCGTTGTCCGGATTTATTG	CTACGCATTTCACCGCTACA
*gtfB*	CACTATCGGCGGTTACGAAT	CAATTTGGAGCAAGTCAGCA
*gtfC*	GATGCTGCAAACTTCGAACA	TATTGACGCTGCGTTTCTTG
*gtfD*	TTGACGGTGTTCGTGTTGAT	AAAGCGATAGGCGCAGTTTA
*ldh*	AAAAACCAGGCGAAACTCGC	CTGAACGCGCATCAACATCA
*atpD*	TGTTGATGGTCTGGGTGAAA	TTTGACGGTCTCCGATAACC

### Cytotoxicity assay on human oral keratinocytes (HOK)

The cytotoxicity of the peptides was measured by the Cell Counting Kit-8 (CCK-8) (Utheim et al., 2016). Briefly, HOK cells (5,000 cells/well) were inoculated in a 96-well plate. After reaching 80% confluence, the cells were exposed to the peptides (3.1, 6.2, 12.5, 25, 50, and 100 μM) for 2 h. In negative control, the peptides were replaced with PBS only. Then CCK-8 solution (10 μL) was added and incubated for 2 h. OD_450_ was measured.

### Anti-caries assay on a caries rat model

The anti-caries effect of GHaR and GHa11R was detected in a caries rat model infected by *S. mutans* (Wang et al., 2018; Tian et al., 2022). Twenty-seven *Sprague Dawley* (SD) male rats aged 18 days (Slyke Jingda Experimental Animal Co., Ltd., China) were fed food (supplemented with 0.1% ampicillin) and water (containing 4,000 U/mL penicillin) for 3 days to sterilize the oral endogenous microorganisms. At the age of 22–24 days, 24 rats were anesthetized by isoflurane and infected orally with 200 μL of *S. mutant* (1 × 10^9^CFU/mL) once daily. Twenty-four hours after the last infection, a sterile cotton swab was used to scratch the oral cavity of the rats, which was then rinsed with PBS. The solution was plated on Mitis Salivarius agar (MSA) containing 20 U/mL bacitracin to confirm colonization. The infected rats were randomly divided into 4 groups with 6 rats in each, including the negative control group (UA159 + PBS), the positive control group (UA159 + CHX), and two peptide-treated groups (UA159 + GHaR and UA159 + GHa11R). Uninfected rats served as the blank group. The rat teeth were evenly brushed with a sterilized cotton swab soaked with 200 μL of PBS, CHX, GHaR or GHa11R three times a week for 8 weeks. All rats were fed Keyes 2,000 # cariogenic diet and purified water containing 5% sucrose. The health status of the rats was recorded. At the age of 81 days, after suffocation by CO_2_, the left and right mandibles were removed. After autoclaving at 121°C for 10 min, the mandibles were cleaned by peeling off the remaining tissue, washed with PBS, and stained overnight with 0.4% murexide. The main groove of the tooth was exposed by a proximal sagittal semi-section and observed under a stereo microscope (M205 FA, LEICA, Germany) to assess the caries level according to a modified Keyes score. An “E” score of 1 indicates that the caries involved only the enamel. A “D” score of 2 indicates that the caries did not exceed 1/4 of the dentin thickness. A “Dm” score of 3 indicates that the range of caries was 1/4–3/4 of the dentin thickness. A “Dx” score of 4 indicates that the caries was more than 3/4 of the dentin thickness. The score for each mandible was the sum of the scores for the first and second molars.

### Statistical analysis

The experiments were performed independently three times in triplicates. The data in the tables and graphs was a representation of one biological replicate and presented as the mean ± standard deviation (SD). The data were analyzed statistically using *t*-test with the GraphPad Prism, with a *P-*value of less than 0.05 considered statistically significant when comparing the treated groups to the control groups.

## Results

### The characteristics and structures of GHa and its derived peptides

In our previous study, we performed sequence alignment on different AMPs and found that the positively charged amino acid lysine (Lys, K) in temporin peptides occurs more frequently than arginine (Arg, R) and histidine (His, H), while in the antibiofilm peptides, R and K are similarly chosen over H (Xie et al., 2019). In order to obtain derived peptides with antibiofilm efficacy, we used R to replace the H at GHa to design GHaR and GHa11R. Compared with GHa, the charge, amphiphilic index (AI) and Boman index (BI) of GHaR and GHa11R increased, and the grand average of hydropathy value (GRAVY) decreased (Table 2). Generally, α-helical AMPs with high amphiphilicity show strong activity; meanwhile their GRAVY is positive, and BI is negative or close to 0 (Saporito et al., 2018). The changes in the physiochemical characteristics of the derived peptides may enhance their antibacterial activities.

**TABLE 2:** The characteristics of temporin-GHa, GHaR, and GHa11R.

Peptides	Amin acid sequence	MW	Charge^a^	pI^a^	AI^a^	BI^b^	GRAVY^b^
GHa	FLQHIIGALGHLF	1464.76	1	7.67	0.32	−1.49	1.315
GHaR	FLQRIIGALGRLF	1502.86	2	12.1	0.47	0.08	1.115
GHa11R	FLQHIIGALGRLF	1483.87	1.5	10.5	0.4	−0.7	1.215

^a^Calculated by Database of Antimicrobial Activity and Structure of Peptides (DBAASP) https://www.dbaasp.org/home. isoelectric point (pI). amphiphilicity index (AI).

^b^Predicted by Antimicrobial Peptide Database (APD3) https://aps.unmc.edu/AP/. Boman index (BI, kcal/moL).

**FIGURE 1:**
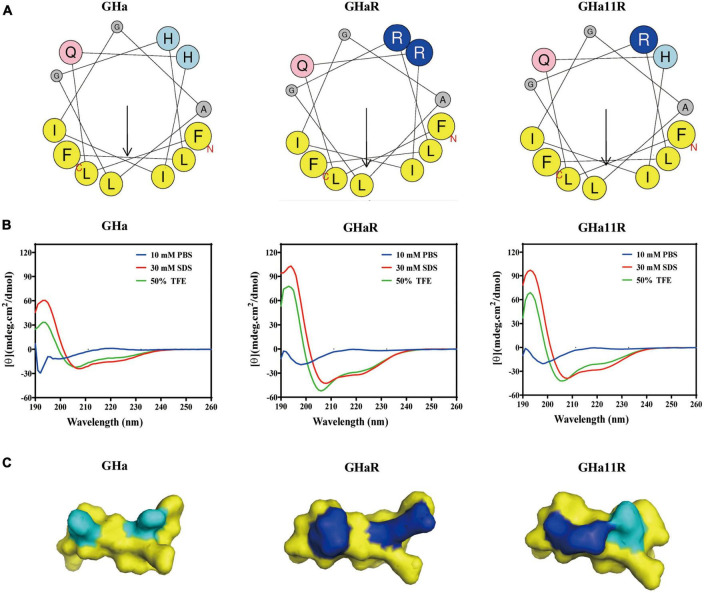
The structures of temporin-GHa, GHaR, and GH11R. **(A)** Heliquest (https://heliquest.ipmc.cnrs.fr/) was used to draw helical wheel projections. The arrows show the hydrophobic moment direction. **(B)** The secondary structures were detected by CD spectroscopy. **(C)** The 3D structures were predicted by PEP-FOLD (http://bioserv.rpbs.univ-paris-diderot.fr/services/PEP-FOLD/) and displayed by PyMol. The light and dark blue represent histidine and arginine residues, respectively.

The structures of the peptides are shown in Figure 1. The spiral wheel of the peptides presented that on the one side the hydrophilic amino acid residues were located, while the hydrophobic residues were arranged on the opposite side, forming the hydrophilic and hydrophobic surfaces, respectively. The CD spectrum revealed that the peptides showed a random-coiled structure in aqueous conditions, while dissolved in a solvent of SDS (30 mM) or TFE (50%), they shown typical α-helical structures, indicating that GHa and the derived peptides had an amphiphile α-helical structure in simulated bacterial membranes. The predicted 3D structure of the peptides is also an α-helix structure. Meanwhile, the increased amphiphilicity of the derived peptides indicated that they have a more stable amphipathic α-helix structure than that of the parent peptide (Abbassi et al., 2013).

### The peptides show stronger antibacterial activity against *S. mutans* than against the tested probiotics

GHa has mild antibacterial activity against *S. mutans* with MIC of 12.5 μM and MBC of 25 μM. By mutating the amino acid residues of GHa, the ability of the derived peptides GHa11R and GHaR to inhibit *S. mutans* growth was improved, with the MIC decreased by 4 times and 8 times, respectively. The antibacterial efficacy of the peptides against several oral etiologic bacteria and probiotics was detected. As shown in Table 3, the peptides also showed good inhibitory activity against the oral pathogenic bacteria *S. sanguinis* and *P. gingivalis*, which are associated with oral infections and cardiovascular diseases. Comparatively, GHa and its derived peptides against pathogenic bacteria had lower MIC/MBC values; however, they showed higher antibacterial concentrations against the tested probiotics. CHX exhibits strong antibacterial efficacy against both pathogenic bacteria and probiotics.

**TABLE 3:** The MIC/MBC of the derived peptides against pathogenic bacteria and probiotics.

Stains	MIC/MBC (μ M)			
	**GHa**	**GHaR**	**GHa11R**	**CHX**
*Streptococcus mutans* UA159	12.5/25	1.6/3.1	3.1/6.2	0.8/1.6
*Streptococcus sanguis* ATCC 10556	1.6/3.1	12.5/12.5	6.2/12.5	0.8/1.6
*Porphyromonas gingivalis* ATCC 33277	12.5/25	3.1/6.2	6.2/12.5	0.8/1.6
*Bifidobacterium adolescentis* ATCC 15703	50/50	6.2/12.5	25/25	3.1/12.5
*Bifidobacterium breve* ATCC 15700	100/200	50/50	100/100	25/25
*Lactobacillus acidophilus* ATCC 4356	25/50	50/100	50/100	25/50

### The peptides exerted antibacterial activity by damaging cell membrane integrity

**FIGURE 2:**
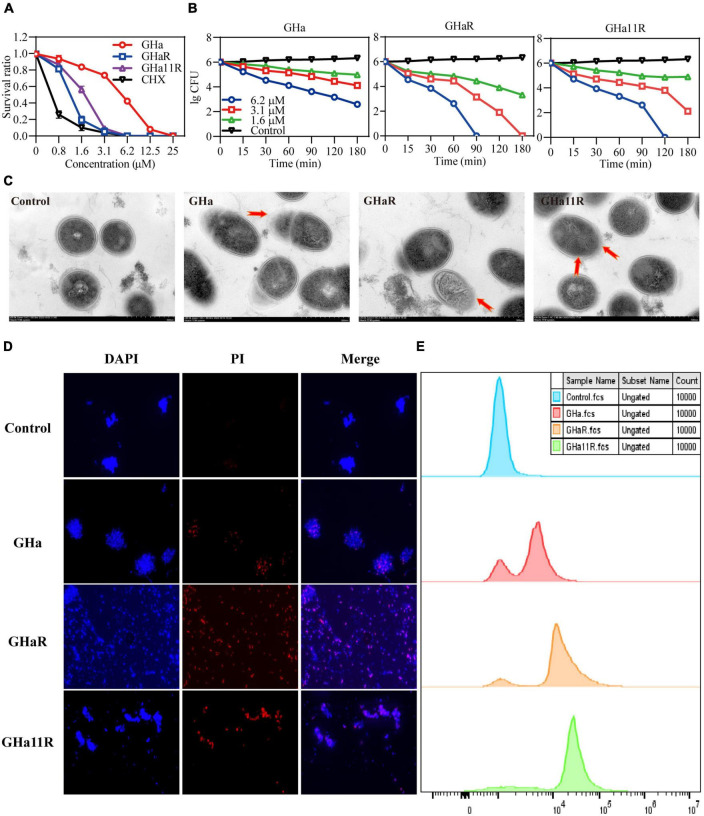
Antibacterial effect of GHa, GHaR, and GHa11R against *S. mutans.* **(A)** The antibacterial activity of GHa, GHaR, and GHa11R against planktonic *S. mutans*. The survival rate was defined as the OD_600_ ratio of *S. mutans* treated with or without the peptides or CHX. **(B)** The time-killing kinetics of GHa, GHaR and GHa11R against *S. mutans* were determined. **(C)** *Streptococcus mutans* exposed to the peptides (12.5 μM ) for 1 h were observed by TEM. **(D)** The cell membrane integrity of *S. mutans* was detected by fluorescence staining. *Streptococcus mutans* treated with GHa, GHaR, or GHa11R were double stained with DAPI and PI. The images were photographed under a fluorescence microscope at a magnification of 40 × . **(E)** The bacteria treated with GHa, GHaR, or GHa11R (1 × MIC) were stained with PI and analyzed by flow cytometry. Bacteria treated with PBS were the negative control.

The bacteriostatic activity of GHa, GHaR, and GHa11R against planktonic *S. mutans* was investigated (Figure 2A). GHaR inhibited *S. mutans* growth at 3.1 μM and GHa11R at 6.2 μM, similar to CHX. The time-killing kinetics demonstrated that the killing effect of these peptides on *S. mutans* was concentration- and time-dependent. After treatment with 6.2 μM GHaR and GHa11R, all bacteria were killed within 90 ∼ 120 min (Figure 2B). The microscopic morphology of *S. mutans* was observed under TEM. As shown in Figure 2C, the bacterial cell membrane was destroyed, resulting in leakage of cellular contents.

### Fluorescence staining revealed the membrane destruction mechanism of the peptides

As shown in Figure 2D, after the bacteria were treated with the peptides (1 × MIC), PI penetrated cell membranes, showing red fluorescence, which indicated that the structure of *S. mutans* cell membranes was destroyed. Flow cytometry was used to further detect the membrane destruction efficiency of the peptides on *S. mutans*. In comparison with the untreated bacteria, after 1 h of treatment with the peptides (1 × MIC), a large number of *S. mutans* stained by PI was detected (Figure 2E).

### The peptides decreased the acidogenicity and aciduricity of *S. mutans*

**FIGURE 3:**
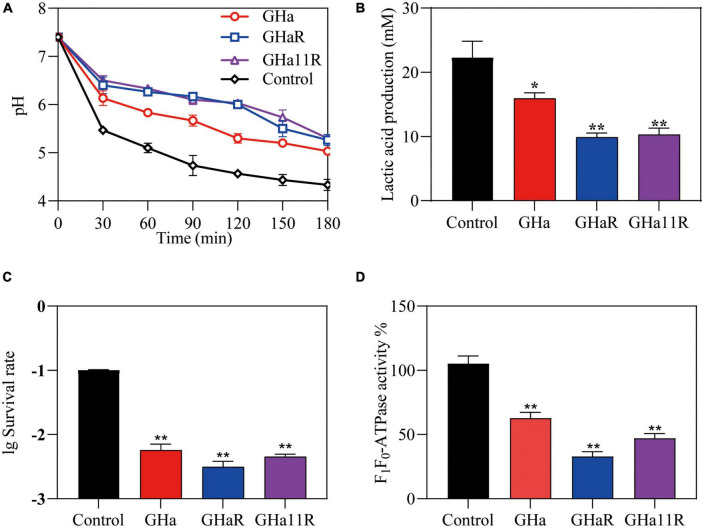
Effects of GHa and its derived peptides on the acidogenicity and aciduricity of *S. mutans.* **(A)** Effects of the peptides on glycolytic pH, **(B)** lactic acid production, and **(C)** aciduricity of *S. mutans* were assayed. **(D)** The F_1_-F_0_ ATPase activity of *S. mutans* after 120 min of treatment with GHa, GHaR and GHa11R was determined. The final concentration of the peptides was 1/2 × MIC. *Represents comparison with the control. *P < 0.05 and **P < 0.01.

As shown in Figure 3A, the pH value of the suspension in the control group declined sharply from 7.4 to 4.5 within 180 min. After exposure to the peptides, the drop in pH was alleviated and decreased to 5.4, indicating that the peptides inhibited the acidogenicity of *S. mutans*, especially GHaR and GHa11R. The effect of the peptides on the acid production of *S. mutans* was further determined (Figure 3B). In comparison with GHa, GHaR and GHa11R reduced lactic acid production by 50% within 180 min. After 3 h incubation under killing pH conditions, 10% of the *S. mutans* bacteria without the peptide treatment still survived, while the treated bacteria decreased to less than 1% of their original population (Figure 3C). Meanwhile, the activity of F_1_-F_0_-ATPase related to acid tolerance of *S. mutans* decreased by 50–70% after being treated with 1/2 × MIC of the peptides for 120 min (Figure 3D).

### The peptides inhibited the attachment of *S. mutans* biofilms

**FIGURE 4:**
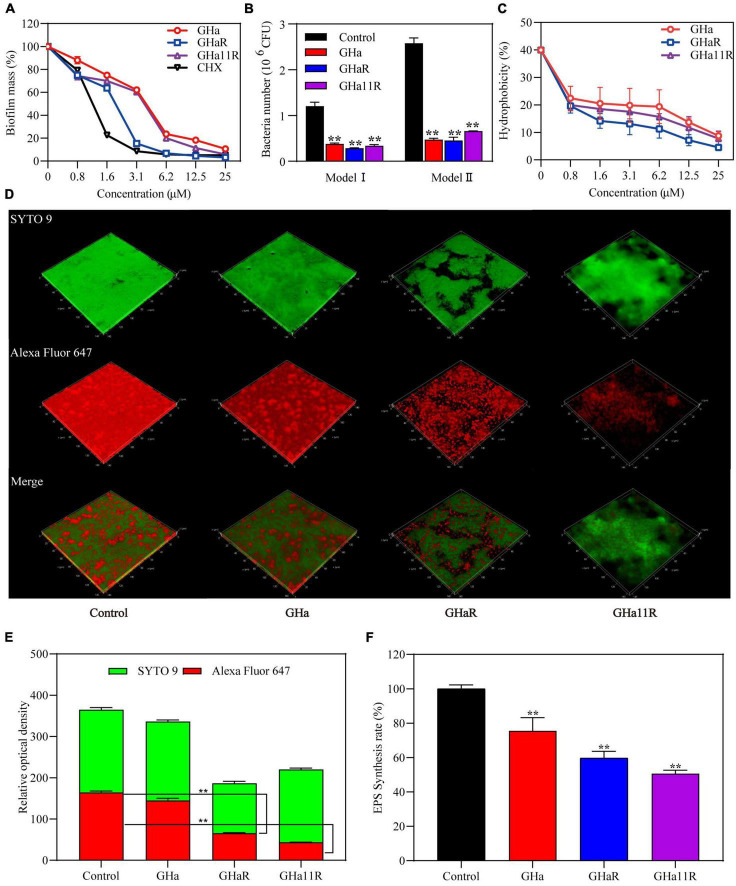
The peptides inhibited *S. mutans* biofilm formation. **(A)** The peptides inhibited the formation of biofilms. After incubation with the peptides for 24 h, the biomass in the biofilms was measured. **(B)** The peptides inhibited the initial bacterial adhesion. Model I represents *S. mutans* adhesion on polyethylene plates, and Model II represents *S. mutans* adhesion on polyethylene plates precoated with artificial saliva. **(C)** The peptides reduced the hydrophobicity of *S. mutans.* **(D)** Observation of *S. mutans* biofilms by CLSM. Representative images of glucan distribution and biofilms are presented. EPS marked with Alexa Fluor 647 are in red; the bacteria labeled with SYTO 9 are in green. Images were taken at 64 × magnification. **(E)** Quantitative data of bacteria and EPS biomass. **(F)** The peptides reduced EPS production in biofilms of *S. mutans*. *Represents comparison with the control. **P < 0.01.

As shown in Figure 4A, when *S. mutans* was incubated with sub-MICs of GHaR and GHa11R for 24 h, more than 25–30% of the bacterial biomass in the *S. mutans* biofilms has been inhibited. Furthermore, we explored the attachment ability of the bacteria to polystyrene plates and saliva-coated polystyrene plates (Figure 4B). After treatment with GHaR and GHa11R, more than 60% of the *S. mutans* attached to polystyrene plates was reduced, indicating that the peptides disturbed the initial adhesion of *S. mutans*, thus inhibiting biofilm formation. Applying saliva to the surface of the polystyrene plate significantly enhanced bacterial attachment, with doubling biofilm formation in the negative control. However, both GHaR and GHa11R still showed effective efficacy in preventing the initial attachment of biofilms.

To explain how the peptides impact the adhesion ability of *S. mutans*, the hydrophobicity of bacterial surfaces exposed to the peptides was investigated. Nearly 40% of *S. mutans* in the negative control was transferred from the aqueous phase to the organic phase. After treatment, approximately 20% of *S. mutans* was transferred, and the percentage decreased dose-dependently (Figure 4C), confirming that the peptides decreased the hydrophobicity of *S. mutans*.

### The peptides reduced the EPS production in biofilms

The glucan labeled by Alexa Fluor 647 can be embedded into EPS during the synthesis of the *S. mutans* biofilm matrix, showing red fluorescence under CLSM. The biofilms in the blank control were tightly packed, and EPS was highly expressed (Figure 4D). Although GHa (1/2 × MIC) showed no significant efficacy on the *S. mutans* biofilms, GHaR and GHa11R destroyed the confluent, tightly packed layers of the biofilms, resulting in scattered clusters of bacteria in the biofilms. As shown in Figure 4E, the EPS in biofilms decreased significantly after exposure to the derived peptides. The inhibitory activity of the peptides on EPS production has been explored (Figure 4F). The water-insoluble EPS production had been decreased by 40–50% after the bacteria were exposed to GHaR and GHa11R.

### The peptides downregulated virulence gene expression in *S. mutans*

**FIGURE 5:**
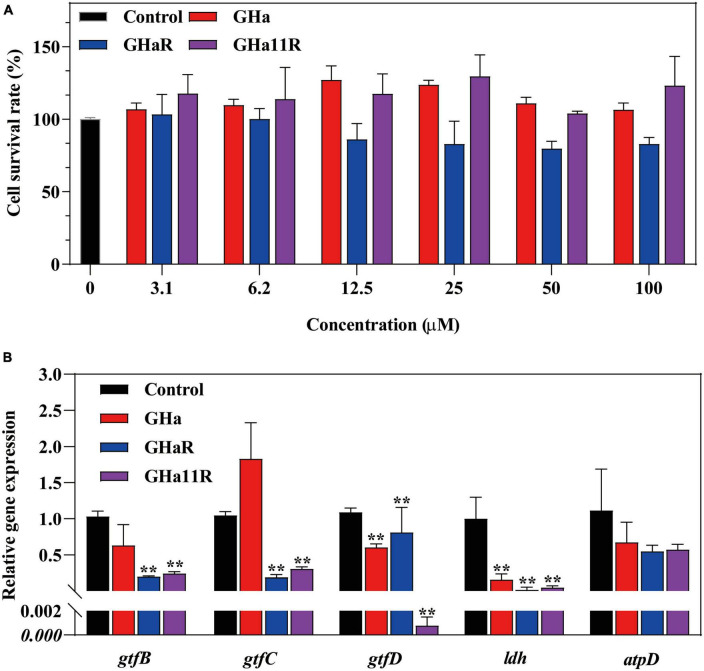
**(A)** The cytotoxicity of GHa, GHaR and GHa11R was tested by CCK-8 assay on human oral keratinocytes. *Represents comparison with the control. **(B)** The effect of GHa, GHaR, and GHa11R on virulence gene expression in *S. mutans* biofilms was tested by qRT-PCR. Gene expression in the peptide-treated *S. mutans* in comparison with the untreated control was quantified. **P < 0.01.

As shown in Figure 5A, the tested genes were downregulated after treatment with sub-MICs of the peptides, especially the *gtfB* and *gtfC*, which encode water-insoluble EPS, and the *ldh*. GHaR and GHa11R reduced the expression of the *gtfB* and *gtfC* genes by 70 to 80%. GHa11R reduced the expression of the *gtfD* gene by more than 99%. For the *ldh* gene, both GHaR and GHa11R reduced the expression of the *gtfD* gene by more than 95%.

### The peptides showed less cytotoxicity

After HOK cells were cocultured with GHa and its derived peptides, cell viability was detected. The results showed that GHa and GHa11R had no toxic effect on HOK cells at a concentration of 100 μM (Figure 5B). GHaR showed a weak inhibitory effect on cell viability at concentrations greater than 12.5 μM. However, the cytotoxicity was not dose dependent, and at least 80% of HOK cells treated with 100 μM GHaR were still alive.

### The peptides showed anti-caries activity in rats

**FIGURE 6:**
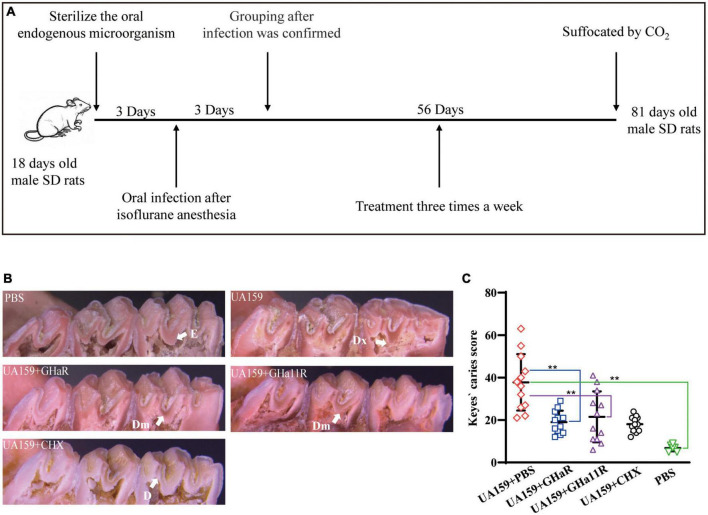
Anti-caries efficacy of the peptides. **(A)** Schematic diagram of the experimental scheme for establishment of the rat caries model. **(B)** Pit and fissure caries lesions on the mandibular molars were observed by stereoscopic microscopy. The arrows indicate the lesions on the sulci surface. E indicates that the caries involved only the enamel. D indicates that the caries did not exceed 1/4 of the dentin thickness. Dm indicates that the range of caries was 1/4–3/4 of the dentin thickness. Dx indicates that the caries was more than 3/4 of the dentin thickness. **(C)** Total Keyes scores of the caries on the first and second mandibular molars were evaluated. *Represents comparison with the control. *P < 0.05 and **P < 0.01.

The derived peptides exhibited better antibacterial efficacy than that of GHa, so we investigated whether GHaR and GHa11R reduce the incidence and severity of tooth decay in a rat caries model (Figure 6A). As shown in Figure 6B, all molars of the rats inoculated with *S. mutans* showed progressive development of dental caries. As indicated by the arrows, the lesions on the sulci surface were significantly more extensive in the control group. The total Keyes scores in mandibular first molar and second molar of the rats were evaluated. The severity of dental caries was decreased by treated with 25 μM GHaR and GHa11R (Figure 6C). GHaR showed effective anti-caries activity as good as CHX.

## Discussion

The oral microbiome consisting of bacteria, fungi, and viruses, is a complicated microbial ecosystem in living organisms (Jiang et al., 2021). The balance of the microbiome is crucial for maintaining oral health; if lost, predominant pathogens may cause oral diseases, such as dental caries, periodontitis and oral mucositis (Lamont et al., 2018). AMPs, the important molecules in the innate immune system, participate in defensing oral microbial infection and controlling resident microbial populations with other innate defense molecules in the oral cavity (Devine, 2003). The cell walls of bacteria, both Gram-positive and Gram-negative, are rich in teichoic acid and lipopolysaccharide, respectively, resulting in negatively charged surfaces, whereas most AMPs have net positive charges due to the basic amino acid residues in their amino acid sequences (Savini et al., 2020). When bacteria are exposed to AMPs, the positive charges of AMPs interact with the negative charges on the bacterial cell surfaces through electrostatic forces, which results in AMPs penetrating the bacterial cells or damaging the cell membranes to exert bactericidal efficacy (Ho et al., 2013).

Histatins are the main AMPs in saliva and are rich in histidine (Griffith et al., 2022), aggregating and integrating into the lipid bilayer of cell membranes and ultimately causing microbial cell death (Lynge Pedersen and Belstrom, 2019). Inspired by the role of histatins in oral defense, we applied the same histidine-rich cationic AMP temporin-GHa to *S. mutans*, and it showed antibacterial activity. It was calculated that the average frequency of occurrence of the positively charged amino acid residues H, R, and K are 2.14%, 5.97%, and 9.72% in 3,569 AMPs in APD3 (Wang et al., 2016), indicating that K and R are the most abundant positively charged amino acids in AMPs. Comparatively, H (pKa 6.5) carries no charge at physiological pH and only a positive charge at low pH (Kacprzyk et al., 2007). The amino acid sequence was changed by substituting R for H, at position 11 to produce the derived peptide GHa11R, which improved the interaction of GHa with the anionic membranes of *S. mutans*. The antibacterial activity of GHa11R (MIC of 3.1 μM) was 4 times higher than that of GHa, and the efficacy of inhibiting biofilm formation was also significantly improved, while the cytotoxicity was similar to that of GHa, without showing a toxic effect on HOK cells at a concentration of 100 μM. To further explore the contribution of the positive charges on the antimicrobial activity and cytotoxicity of GHa, two H amino acid residues on GHa were substituted by R, leading to GHaR, whose antibacterial performance was also enhanced with an MIC of 1.6 μM, and the bactericidal efficacy on *S. mutans* was faster than that of GHa11R. Although the cytotoxicity of GHaR to HOK cells was increased, at least 80% of HOK cells survived at a concentration of 100 μM. We confirmed that the increase of the net positive charges in peptides improves the activity of AMPs and correspondingly enhances their toxicity to cells (Ramezanzadeh et al., 2021).

The mechanism of action of the peptides on *S. mutans* was determined. Both DAPI and PI can bind to DNA, while DAPI passes through intact cell membranes and PI penetrates damaged cell membranes. *S. mutans* treated with GHa and its derived peptides showed bright red fluorescence after double staining with PI and DAPI, indicating that the membrane permeability was increased and the membrane integrity was damaged. The results were further confirmed by flow cytometry assay. The clear and smooth cell surface edge of the peptide-treated bacteria was lost; the large-scale damage of the membranes and the rupture of the membrane surfaces were clearly observed through SEM. Therefore, GHa and its derived peptides exerted their killing effect on *S. mutans* by increasing the cell membrane permeability, leading to the destruction of their integrity and leakage of intracellular components.

The cariogenic potential of *S. mutans* is regulated mainly by its virulence factor related genes (Shanmugam et al., 2020). Through our research, *S. mutans* carotenoid virulence factors were blocked by GHa and its derived peptides, which reduced the bacteria’s capacity for acidogenicity and aciduricity, decreased the production of EPS, and prevented the growth of biofilms. One of the main pathogenic features of *S. mutans* is lactic acid production, in which LDH encoded by the *ldh* gene, plays a key role (Allen et al., 2021). GHa, GHaR, and GHa11R significantly inhibited the expression of the *ldh* gene, thereby reducing lactic acid production and alleviating the pH drop in *S. mutans*. Acid resistance and production of *S. mutans* are highly correlated, which determines whether *S. mutans* can survive in the acidic environment created by itself. F_1_-F_0_ ATPase regulates and maintains the acid-base characteristics by participating in proton transport, which reflects the acid resistance level of *S. mutans* (Sekiya et al., 2019). The expression of the *atpD* gene encoding the α subunit of the F_1_-F_0_ ATPase was downregulated by the peptides, resulting in a reduction in the aciduricity of *S. mutans*. In general, GHa and its derived peptides showed the ability to reduce cariogenic virulence of *S. mutans* by inhibiting the expression of acidogenicity genes and aciduricity genes of *S. mutans*.

During biofilm formation, *S. mutans* initially attaches to tooth surfaces, followed by the production of EPS, which can be utilized as a matrix to encourage the aggregation of additional pathogen, ultimately leading to the creation of multispecies biofilms (Zhang et al., 2021). We found that the adhesion ability of *S. mutans* exposed to the peptides was weakened, which reduced the initial bacterial attachment and blocked or delayed the biofilm formation at the initial stage. *S. mutans* synthesizes EPS by Gtfs converting sucrose into sticky gelatinous extracellular glucan, providing a perfect matrix for other oral microorganisms to attach (Ma et al., 2015). There are three Gtfs in *S. mutans*, namely, GtfB, GtfC, and GtfD (Bowen and Koo, 2011). Water-insoluble glucans are produced by GtfB, and soluble glucans synthesized by GtfD mainly; while GtfC catalyzes both soluble and insoluble glucans (Ma et al., 2021). Therefore, downregulating the expression of Gtfs is a significant way to inhibit the initial biofilm formation. The expression of the *gtfB*, *gtfC* and *gtfD* genes in *S. mutans* was downregulated significantly by GHa, GHaR, and GHa11R, especially that of *gtfB* and *gtfC*, which decreased by nearly 80%. GHa and its derived peptides reduced EPS production by 20–40% at sub-MICs. Downregulation of Gtfs expression by the peptides reduced the production of EPS and impaired subsequent adhesion and biofilm formation. We further observed EPS in the biofilms using CLSM and found that in the blank control, *S. mutans* formed dense and thick biofilms, and many bacteria clustered together in it; EPS expression was highly coincident with the biofilms by labeling with Alexa Fluor 647 glucan *in situ*. In comparison with GHa, the derived peptides GHaR and GHa11R showed better inhibitory efficacy of EPS synthesis, especially GHaR, which significantly reduced the accumulation of *S. mutans* biofilms and decreased EPS production; although GHa11R showed mild inhibitory activity on *S. mutans* aggregation, the production of EPS was reduced significantly. In conclusion, GHaR and GHa11R exhibited different performances in inhibiting biofilm formation. GHaR showed a stronger effect in reducing the initial adhesion and hydrophobicity of *S. mutans* and subsequently inhibited the accumulation of biofilms. GHa11R reduced the expression of EPS-related genes, showing a stronger effect in reducing the EPS synthesis.

The safety of AMPs has been a concern in their application. GHa and GHa11R were not toxic to HOK cells at a concentration of 100 μM; although GHaR showed slight cytotoxicity, the IC_50_ was more than 100 μM, indicating that GHa and its derived peptides have excellent biocompatibility. We tested the effect of the peptides on other oral pathogenic bacteria and found that they showed strong antibacterial activity against *S. sanguinis*, which plays an important role in the formation of upper and subgingival plaques, and *P. gingivalis*, the main pathogenic bacteria of periodontal disease. In addition, we tested the inhibitory ability of GHa and its derived peptides against several common probiotic bacteria, such as *Bifidobacterium* and *Lactobacillus*, and the results demonstrated that the peptides showed much higher inhibitory concentrations for probiotic bacteria than oral pathogenic bacteria. These results indicated that the peptides at the therapeutic concentration selectively exerted bactericidal efficacy on oral pathogenic bacteria, which has positive significance for the application of GHa and its derivatives to oral diseases. We evaluated the *in vivo* effects of GHaR and GHa11R in a rat caries model. The results of Keyes scores showed that compared with the model group, caries development significantly slowed in the GHaR and GHa11R groups, and the severity of caries was controlled. The results demonstrated that GHaR and GHa11R had the potential to relieve caries related to *S. mutans* in the oral cavity of rats.

Here, we designed temporin-GHa-derived peptides GHaR and GHa11R, which showed selectively strong antibacterial activity against oral etiologic bacteria, including *S. mutans*, *S. sanguinis*, and *P. gingivalis*. The derived peptides exerted bactericidal efficacy on *S. mutans* plankton by interrupting the integrity and permeability of cell membranes, resulting in intracellular component leakage. They also showed antibiofilm activity against *S. mutans* by reducing surface hydrophobicity, disturbing the initial adhesion, and inhibiting virulence factor production, including lactic acid and EPS. GHaR and GHa11R delay the development of dental caries associated with *S. mutans* in the oral cavity of rats, showing promising application in the treatment of *S. mutans* infections.

## Data availability statement

The original contributions presented in this study are included in the article/supplementary material, further inquiries can be directed to the corresponding author.

## Ethics statement

The animal study was approved by the Animal Ethics Committee of Hainan University. The study was conducted in accordance with the local legislation and institutional requirements.

## Author contributions

SJ: Writing–original draft, Writing–review and editing, Conceptualization. YZ: Methodology, Writing–review and editing. TZ: Methodology, Writing–review and editing. XJ: Methodology, Writing–review and editing. RZ: Methodology, Writing–review and editing. SW: Writing–review and editing. RW: Writing–review and editing. YS: Writing–review and editing. LL: Writing–review and editing. JL: Writing–review and editing. WH: Writing–review and editing. DZ: Writing–review and editing. MW: Writing–review and editing. YZ: Writing–original draft, Writing–review and editing.
